# Possible ameliorative effects of antioxidants on propionic acid / clindamycin - induced neurotoxicity in Syrian hamsters

**DOI:** 10.1186/1757-4749-5-32

**Published:** 2013-11-04

**Authors:** Afaf El-Ansary, Ghada Shaker, Nikhat J Siddiqi, Laila Y Al-Ayadhi

**Affiliations:** 1 Biochemistry Department, Science College, King Saud University, P.O Box 22452, 11495, Riyadh, Saudi Arabia; 2 Department of Microbiology and Immunology, College of Pharmacy, Zagazig University, Zagazig, Egypt; 3 Department of Physiology, Faculty of Medicine, King Saud University, Riyadh, Saudi Arabia; 4 Autism Research and Treatment Unit, Riyadh, Saudi Arabia; 5 Shaik AL-Amodi Autism Research Chair, King Saud University, Riyadh, Saudi Arabia; 6 Therapuetic Chemistry Department, National Research Centre, Dokki, Guiza, Egypt

**Keywords:** Autism, Clindamycin, Propionic acid, Carnosine, Carnitine, Cortex, Medulla

## Abstract

**Background:**

Propionic acid (PA) found in some foods and formed as a metabolic product of gut bacteria has been reported to mimic/mediate the effects of autism. The present study was undertaken to compare the effect of orally administered PA with that of clindamycin-induced PA-microbial producers in inducing persistent biochemical autistic features in hamsters. The neuroprotective potency of carnosine and carnitine supplements against PA toxicity was also investigated.

**Methods:**

The following groups were studied. 1. Control group, which received phosphate buffered saline orally, 2. Propionic acid treated group which were given PA at a dose of 250 mg/kg body weight/day for 3 days orally, 3. Clindamycin treated group which received a single dose of the antibiotic orogastrically at a dose of 30 mg/kg on the day of the experiment, 4. Carnosine-treated group which were given carnosine at a dose of 10 mg/kg body weight/day orally for one week, 5. Carnitine treated group given 50 mg/kg body weight/day carnitine orally daily for one week. Group 6. Carnosine followed by PA, Group 7. Carnitine followed by PA. Dopamine, adrenaline and noradrenaline, serotonin and Gamma amino-butyric acid (GABA) were measured in the cortex and medulla of the nine studied groups.

**Results:**

PA administration caused significant decrease in the neurotransmitters in the brains of treated hamsters while clindamycin caused a significant decrease only in dopamine in hamster brains (cortex and medulla) and GABA in the cerebral cortex of the treated hamsters. Administration of carnosine and carnitine which are known antioxidants caused no significant changes in the levels of neurotransmitters when administered alone to hamsters. However when administered with PA both carnosine and carnitine restored the altered neurotransmitters to near normal levels.

**Conclusion:**

Carnosine and carnitine may be used as supplements to protect against PA neurotoxicity.

## Introduction

Propionic acid (PA) occurs naturally in a few food products; for example PA is present in low quantities in milk and relatively higher levels in dairy products such as yogurt and cheese, obviously due to bacterial fermentation, mostly by propionibacteria [1,2]. The food sources however have a minor contribution in the PA levels in the body [3]. In the colon, PA is produced by fermentation of polysaccharides, oligosaccharides, long-chain fatty acids, protein, peptides and glycoprotein precursors by the anaerobic colonic microbiota, although in quantitative terms undigested carbohydrates, such as dietary fibers and resistant starch, represent the major source for PA production [3,4].

PA is a short chain fatty acid formed endogenously in the human body as an intermidiate of fatty acid metabolism and a metabolic end product of enteric gut bacteria such as clostridia and propionibacteria [3,5-7]. Recent studies of El-Ansary et al. [8] have demonstrated that PA administration to rats caused an increased generation of oxidative stress which was accompanied by impairment of antioxidant defense indices.

Autism is a disorder of neural development which includes development deficiencies of language and social interaction skills, appearance of repetitive and disordered movements [9] hyperactivity, sensory disturbances, restricted interests and sometimes self injury [10,11]. Intraventricular infusions of PA have been shown to cause behavioral and brain abnormalities in rats similar to those seen in humans suffering from autism [12-14]. Gut microbes have long been suspected to play a role in autism spectrum disorders (ASD) [15]. Gut microbiota also play an important role in metabolism of xenobiotics, modification of drugs etc., [16]. Clindamycin has been shown to cause loss of majority of normal caecal flora and induction of PA producing bacteria among which is *Clostridia* species [17]. In this study hamsters were treated with the antibiotic clindamycin to see if its effects were similar to that of exogenous PA which has been implicated in autism. Attempts were also made to understand the neurochemical effects of PA and induced PA production either alone or in combination with carnosine, carnitine as potent antioxidants.

## Material and methods

### Chemicals

Propionic acid, carnosine and carnitine were of analytical grade products of Sigma-Aldrich.

Clindamycin (Cleocin phosphate) was obtained from Pharmacia Co.,Peapack, NJ, USA).

### Animals

A total of 54 young male golden Syrian hamsters weighing about 80–100 grams were used in the present study. Animals were randomly into seven groups, each consisting of 6 animals. The groups studied included 1. Control group, which received phosphate buffered saline orally, 2. Propionic acid treated group which were given PA at a dose of 250 mg/Kg body weight/day for 3 days orally [18], 3. Clindamycin treated group which received a single dose of the antibiotic orogastrically at a dose of 30 mg/kg on the day of the experiment, 4. Carnosine-treated group were given carnosine at a dose of 10 mg/kg body weight/day orally for one week, 5. Carnitine treated group were given 50 mg/kg body weight/day carnitine orally daily for one week. The protected groups included Group 6. Carnosine followed by PA, Group 7. Carnitine followed by PA. The carnosine and carnitine protected group were given the same doses of the respective compounds for one week followed by PA for three days. All the groups were kept at controlled temperature (21 ± 1°C) with ad libtium access to food and water. The experiments were performed in accordance with national animal care guidelines approved by the Animal Ethics Committee, King Saud University, Riyadh.

### Brain tissue preparation

At the end of experiment, hamsters were killed by carbon dioxide asphyxiation. The brains were dissected out and washed in saline. Cortex and medulla were separated. The tissues were homogenized in double distilled water and the homogenates were stored at −80°C until used.

### Catecholamines assay

Dopamine, adrenaline and noradrenaline were extracted from rat brain by using a cis-diol-specific affinity gel, acylated and then derivatized enzymatically. Quantitavive assay of the three neurotransmitters were performed using ELISA kit, a product of Immuno Biological Laboratories (IBL) using dopamine, adrenaline and noradrenaline antiserum provided. Sensitivities for adrenalin of 0.3 ng/mL, for noradrenaline of 0.6 ng/mL and for dopamine of 5 ng/mL for diluted sample were recorded.

### Assay of serotonin

Serotonin was measured using an ELISA kit, a product of Immunology Biological Laboratories (IBL). Brain homogenate (derivatization of serotonin to N-acylserotonin) was done by sample dilution and incubation of the respective sample with the acylation reagent. The assay procedure followed the basic principle of competitive ELISA whereby there is competition between a biotinylated and a non-biotinylated antigen for a fixed number of antibody binding sites. The amount of biotinylated antigen bound to the antibody is inversely proportional to the N-acylserotonin concentration of the sample. When the system is in equilibrium, the free biotinylated antigen is removed by a washing step and the antibody bound biotinylated antigen is determined by use of anti-biotin alkaline phosphatase as marker and p-nitrophenyl phosphate as substrate. Quantification of unknowns was achieved by comparing the enzymatic activity of samples with a response curve prepared by using known standards.

### Assay of gamma amino-butyric acid (GABA)

Quantitative determination of GABA was done using ELISA immunoassay kit, a product of ALPCO. 300 μL of diluted standards, controls and undiluted samples were placed into the appropriate wells of the extraction plate. 300 μL of the diluent was added to all wells, covered with adhesive foil and shook for 30 min at room temperature (20-25°C) on a shaker (600 rpm). Two washing cycles were performed, after which 250 μl elution buffer was placed into the appropriate wells of the extraction plate, covered and shook followed by addition of 100 μL of the extract for subsequent derivatization. 10 μL of NaOH was added to all the wells followed by 50 μL of the equalizing reagent (fresh prepared before assay) and shook for 1 min on a shaker (600 rpm). 10 μL of the D-reagent was added into all wells, incubated for 2 hours at (20-25°C) and then 150 μL Q-buffer was added into all wells, incubated for 10 min at RT (20-25°C) on a shaker (approx. 600 rpm). 25 μL of the derivative was used for the subsequent ELISA.

### Statistical analysis

A computer SPSS program was used, and the results were expressed as mean ± SD (n = 6). Comparisons were made by the one-way ANOVA between the control and treated groups. Dunnett’s test was used to compare between the groups. Receiver operating characteristic (ROC) analysis was done. Area under the curve, specificity and sensitivity were calculated.

## Results and discussion

Autism spectrum disorders (ASD) comprise a complex and heterogeneous group of conditions that include autism, Rett and Asperger syndromes, and pervasive developmental disorder-otherwise non specified [19]. The main clinical features of ASD are stereotypic behaviors and marked impairment in communication, social skills and cognition [20,21]. Animal models help us understand the mechanism of disease process and the environmental factors which serve as trigger for the disease process. Studies of MacFabe et al. [12] have demonstrated PA injection in rats provides a suitable animal model to study ASD. Moreover, there are a series of inherited and acquired conditions which lead to elevations of PA and other short chain fatty acids and these are related to developmental delay, seizures and gastrointestinal symptoms, resembling some aspects of autism [22,23]. Thus, PA may be a putative link between dietary or enterobacterially derived metabolites along with genetic predisposition and subsequent features of autism [8].

**Figure 1:**
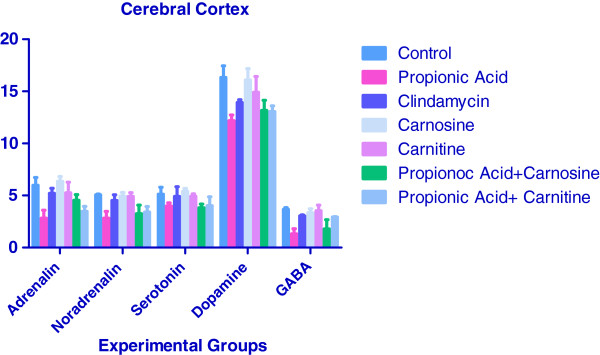
**Effect of propionic acid, antibiotic clindamycin, carnosine, carnitine alone and in combination on the neurotransmitter levels in the cerebral cortex of hamsters.** Concentrations of various neurotransmitters were ng/ml.

Figure 1 shows the effect of PA, antibiotic clindamycin, carnosine, carnitine alone and with PA on the neurotransmitter levels in the cerebral cortex of hamsters. Administration of PA alone caused a significant decrease in the levels of neurotransmitters viz., adrenalin, noradrenalin, serotonin, dopamine and GABA by 53, 44, 22, 25 and 64% respectively when compared to the control group (P <0.05). This is in accordance with the earlier report of El-Ansary et al. [8] where a significant decrease in neurotransmitters was observed in rats treated with PA. The decrease in the levels of neurotransmitters may be due to affect of PA on the transcription of the tyrosine hydroxylase gene encoding the rate-limiting enzyme in the biosynthesis of the neurotransmitters dopamine, norepinephrine and epinephrine. Short chain fatty acids like butyrate are known to alter tyrosine hydroxylase gene expression [24]. Norepinephrine (also known as noradrenaline) is a catecholamine that is synthesized from dopamine through the action of the enzyme dopamine beta-hydroxylase [25]. Noradrenergic activity has been assessed in autism via measurement of norepinephrine (NE) and its central and/or peripheral metabolites in blood, urine, and CSF [25]. Various studies suggest little role for NE in the expression and etiology of autistic disorder [25]. In the present study PA decreased the levels of adrenalin and norepinephrine in hamster brains while carnosine/carnitine with PA showed a tendency towards restoration to normal levels.

Antibiotics are commonly used pharmaceuticals. While most of them are well tolerated, they do have side effects the most common being diarrhea. The neurotoxic effects of antibiotics are not well recognized [26]. Therefore there are chances of neurotoxic effects of antibiotics being confused with different neurological conditions [26].

Clindamycin administration has previously been shown to markedly suppress the anaerobic microbial community including members of the *Bacteroides* group, which account for ∼ 30% of the intestinal bacterial flora [2,27,28]. The change in the intestinal flora may interfere with intestinal enzyme DPP-IV (dipeptydal peptidase), which catalyzes the breakdown of proteins into amino acids, and has been hypothesized as a contributing factor to autism [29]. This is due to the fact that this enzyme is necessary to breakdown peptides into amino acids which are precursors of neurotransmitters.

Though clindamycin is not known have major neurotoxic effects [26] in the present study treatment of hamsters with antibiotic alone caused a significant decrease in dopamine by 15% (P >0.05) and GABA by 16% (P <0.05) in the cortex when compared to the cerebral cortex of control group of rats. Though the exact cause of decrease of these neurotransmitters is not known, it may be due to inhibition of monoamine oxidase [30]. Carnosine is a an antioxidant dipeptide consisting of amino acids histidine and alanine. L-Carnosine is a highly effective anti-aging nutrient that it possesses powerful antioxidant, free radical scavenging and neurotransmitter properties. Carnosine inhibits the formation of carbonyl groups, thereby reducing the formation of abnormal proteins. L-Carnosine extends maximum cell division capacity, protects against DNA oxidation, blocks glycosylation and reduces Advanced Glycation Endproducts, as well as acts as a cell membrane stabilizer and an intracellular buffer [31]. L-Carnosine has recently been shown to possess a tremendous potential for improving language and behavior in children diagnosed with ASDs [31]. In the present study carnosine treatment caused no significant change in the levels of neurotransmitters studied (P >0.05). Similarly carnitine treatment alone caused no significant change in the levels of neurotransmitters studied (P >0.05). Earlier studies [32,33] have found that acetyl-L-carnitine treatment increases noradrenalin and serotonin levels in brains of treated mice. This was accompanied by decrease in the levels of GABA in the brains of treated mice [33]. Co administration of propionic acid and carnosine to hamsters caused an increase in adrenalin by 60% (P <0.05), dopamine by 8% (P <0.05) and in GABA by 35% (P <0.01) when compared to the group treated with propionic acid alone in the cortex of treated hamsters.

Treatment of propionic acid and carnitine also caused an increase in adrenalin by 23% (P < 0.01), noradrenalin by 20% (P < 0.01), dopamine by 7% (P <0.05) and GABA by 120% (P <0.01) when compared to the group treated with propionic acid alone.

**Figure 2:**
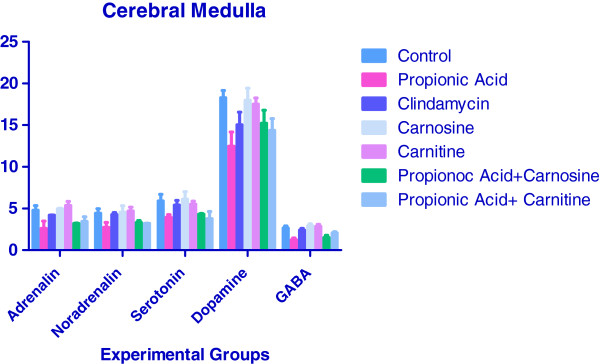
**Effect of propionic acid, antibiotic clindamycin, carnosine, carnitine alone and in combination on the neurotransmitter levels in the medulla of hamsters.** Concentrations of various neurotransmitters were ng/ml.

Figure 2 shows the effect of propionic acid, antibiotic clindamycin, carnosine, carnitine alone and with propionic acid on the neurotransmitter levels in the cerebral medulla of hamsters. Treatment of hamsters with propionic acid caused a decrease in adrenalin by 45% (P <0.05), noradrenalin by 38% (P <0.05), serotonin by 33% (P <0.05), dopamine by 32% (P <0.01) and GABA by 54% (P <0.01) when compared to cerebral medulla of control group. This may be due to the effect of PA on tyrosine hydroxylase [24]. Glutamate and gamma-aminobutyric acid (GABA) are the two neurotransmitters that are linked to widespread synaptic communication in the central nervous system [25]. Glutamate is the principal excitatory neurotransmitter in the brain and spinal cord, whereas GABA is responsible for most of the inhibitory communication in the brain [34,35]. These substances are widely produced in the central nervous system by the cells’ metabolic processes, and their effects are widespread. There are few areas in the brain that do not receive input from glutamate and GABA [34]. Research in these two neurotransmitters has been tightly-linked, as GABA is converted from glutamate by the enzyme glutamic acid decarboxylase (GAD). GAD is the rate-limiting step of the synthesis of GABA [25]. Fatemi et al., 2008 found that this enzyme was reduced by 48–61% in parietal and cerebellar areas of brains of individuals with autism when compared to controls [36]. These differences were statistically significant, and provide an insight into the abnormal levels of GABA in brain during autism. Administration of antibiotic clindamycin to hamsters caused significant decrease only in dopamine levels by 18% (P <0.05) when compared to control hamsters medulla. This effect is parallel to the effect of PA on hamster cortex. As in the case of cortex, carnosine and carnitine treatments alone to hamsters caused no significant change in the levels of any of neurotransmitters (P >0.05). Propionic acid treatment along with carnosine caused significant increases in the levels of adrenalin by 22% (P <0.01), noradrenalin by 22% (P <0.05), dopamine by 22% (P <0.05) and GABA by 24% (P <0.01) when compared to that in the medulla of propionic acid treated hamsters. Propionic acid and carnitine treatment to hamsters caused an increase in adrenalin by 32% (P <0.01), noradrenalin by 3% (P <0.05), dopamine by 15% (P <0.05) and a decrease in serotonin by 5% (P <0.05) when compared to the group of hamsters treated with propionic acid alone. Our earlier studies have also demonstrated the toxic effect of PA through DNA damage and the protective effects of carnosine and carnitine on PA induced DNA damage [37].

ROC analysis showed satisfactory values of area under the curve, sensitivity and specificity. This helps to suggest that the measured neurotransmitters could be used as biomarkers for PA and bacterial overgrowth neurotoxicity and as predictive markers to follow the efficacy of both carnosine and carnitine in ameliorating the toxic effects related to PA.

## Conclusion

The results of the present study lead us to speculate that PA may play a role in ASD by interfering with the neurotransmitters. Carnosine and carnitine which are known antioxidants cause no significant changes in the levels of neurotransmitters when administered alone to hamsters. However when administered with PA both carnosine and carnitine tended to restore the altered levels of neurotransmitters to near normal levels. Therefore carnosine and carnitine may be used as antioxidants as supplements to protect against PA neurotoxicity.

## Competing interests

The authors declare that they have no competing interests.

## Authors’ contributions

AE: Designed the work and co-drafted the manuscript, GS: Performed the microbiological work, NJS: Drafted the manuscript, LA: Did the statistical analysis and revised the manuscript. All authors read and approved the final manuscript.
